# A promising new cancer marker: Long noncoding RNA EGFR-AS1

**DOI:** 10.3389/fonc.2023.1130472

**Published:** 2023-02-24

**Authors:** Danhua Zhu, Xiaoxi Ouyang, Yanhong Zhang, Xiaopeng Yu, Kunkai Su, Lanjuan Li

**Affiliations:** State Key Laboratory for Diagnosis and Treatment of Infectious Diseases, National Clinical Research Center for Infectious Diseases, National Medical Center for Infectious Diseases, Collaborative Innovation Center for Diagnosis and Treatment of Infectious Diseases, The First Affiliated Hospital, Zhejiang University School of Medicine, Hangzhou, China

**Keywords:** lncRNA, EGFR-AS1, expression, functions, clinical values

## Abstract

Cancer consists of a group of diseases with the salient properties of an uncontrolled cell cycle, metastasis, and evasion of the immune response, mainly driven by the genomic instability of somatic cells and the physicochemical environment. Long noncoding RNAs (lncRNAs) are defined as noncoding RNAs with a length of more than 200 nucleotides. LncRNA dysregulation participates in diverse disease types and is tightly associated with patient clinical features, such as age, disease stage, and prognosis. In addition, an increasing number of lncRNAs have been confirmed to regulate a series of biological and pathological processes through numerous mechanisms. The lncRNA epidermal growth factor receptor antisense RNA 1 (EGFR-AS1) was recently discovered to be aberrantly expressed in many types of diseases, particularly in cancers. A high level of EGFR-AS1 was demonstrated to correlate with multiple patient clinical characteristics. More importantly, EGFR-AS1 was found to be involved in the mediation of various cellular activities, including cell proliferation, invasion, migration, chemosensitivity, and stemness. Therefore, EGFR-AS1 is a promising marker for cancer management. In this review, we introduce the expression profile, molecular mechanisms, biological functions, and clinical value of EGFR-AS1 in cancers.

## Introduction

Cancer has been a leading cause of disease-related mortality worldwide in the last decade, with high morbidity and poor functional outcomes (1–5). Despite the existence of accurate biopsy-based diagnosis methods and advances in anticancer drug development, cancer recurrence and poor prognosis still cannot be avoided (6–9). The molecular pathogenesis of various cancers has been persistently investigated and our understanding has expanded, and the related findings help to accurately diagnose cancer and personalize treatment (10–13).

Increasing evidences have shown that noncoding RNAs (ncRNAs) play essential roles in tumor progression (14, 15). Long noncoding RNAs (lncRNAs) are a class of noncoding RNAs with a length of over 200 nucleotides (16–18). An increasing number of lncRNA structures and functions have been discovered in recent years (19, 20). Various lncRNAs have been discovered to be abnormally expressed in many human diseases, especially cancers (21–24). Moreover, emerging studies have revealed lncRNA involvement in various cellular processes, including the cell cycle, differentiation, and chromatin modification (25–28). Functional experiments confirmed that lncRNAs drive their roles through diverse molecular mechanisms, including interactions with proteins, binding to miRNAs, and combining with chromatin-modifying complexes (29–33). In view of their diverse signatures, lncRNAs exhibit a specific advantage in classifying different types of disease as biomarkers for diagnosis, prognosis, and treatment response (34–36).

**Figure 1:**
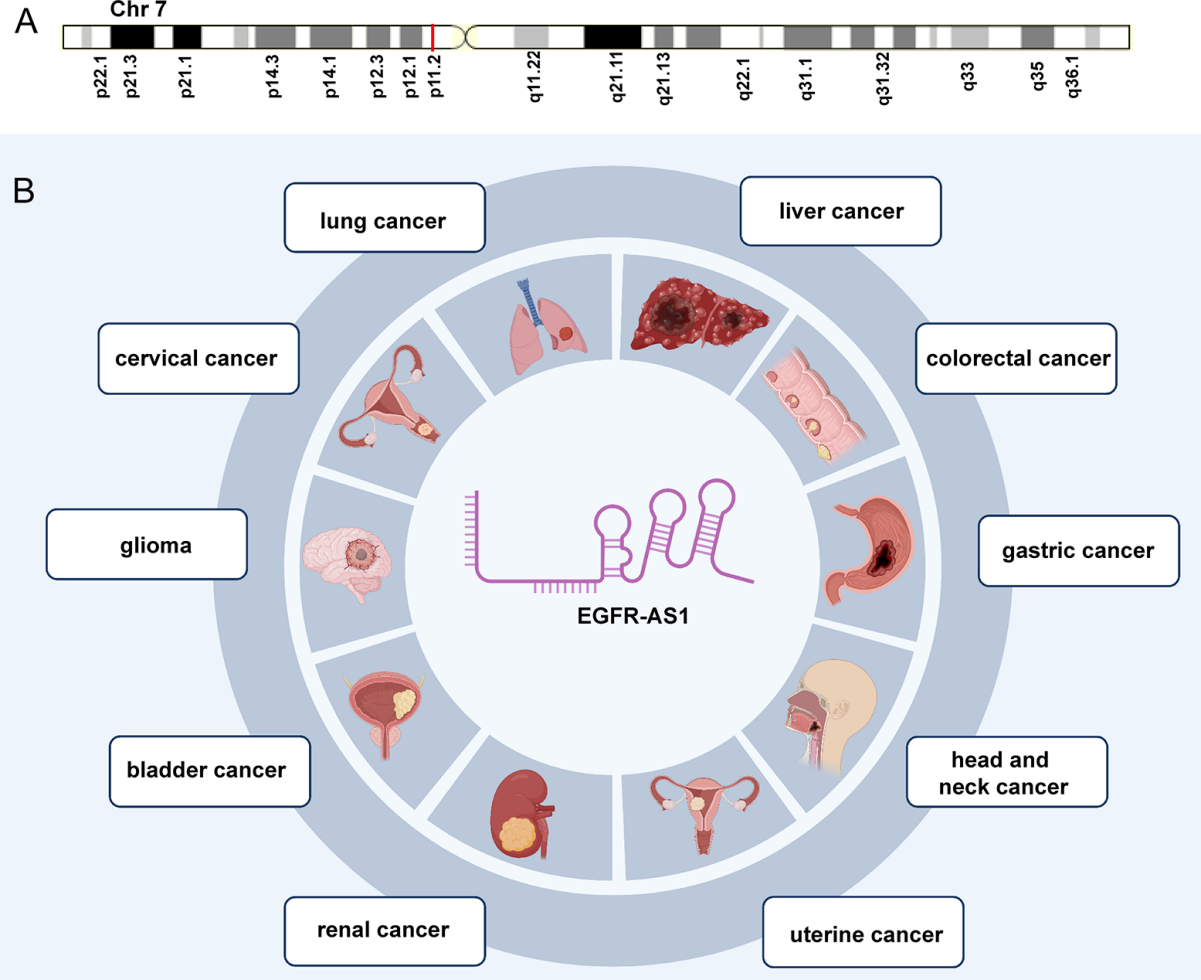
The expression and potential roles in cancer types. **(A)** Chromosomal location of EGFR-AS1. **(B)** EGFR-AS1 was overexpressed in lung cancer, cervical cancer, glioma, bladder cancer, kidney cancer, head and neck cancer, gastric cancer, colorectal cancer, liver cancer, and uterine cancer.

As a 2.821-kb transcript from the antisense chain of epidermal growth factor receptor (EGFR), lncRNA EGFR antisense RNA 1 (EGFR-AS1) belongs to the family of receptor tyrosine kinases ErbB and is located on human chromosome 7p11.2 (chr7:55179750-55188934 according to hg38) (**Figure 1A**) (Images in **Figure 1A** from the GeneCards website, https://www.genecards.org/cgi-bin/carddisp.pl?gene=EGFR-AS1&keywords=EGFR-AS1). Numerous articles have pointed out that the aberrant expression of EGFR-AS1 is involved in the progression of diverse diseases, such as lung cancer (37–40), cervical cancer (41, 42), glioma (43, 44), bladder cancer (45, 46), kidney cancer (47, 48), head and neck cancer (49–51), gastric cancer (52), colorectal cancer (53), liver cancer (54), uterine cancer (55), preeclampsia (56), gestational diabetes mellitus (57), and cryptorchidism (58). EGFR-AS1 is also crucial in physiological processes, including cell migration, invasion, multiplication, and drug sensitivity. High EGFR-AS1 is closely correlated with a poor prognosis and adverse clinical features, such as malignant lymphatic metastasis status, advanced tumor stage, and large tumor size. Moreover, EGFR-AS1 was recommended to be utilized to evaluate cancer prognosis and the efficacy of chemotherapies and has promising diagnostic performance in different diseases. This review mainly focused on the correlation of EGFR-AS1 expression with the clinicopathological features and prognosis of patients, its functions in regulating cellular processes, and its promising clinical applications.

## Expression and functions of EGFR-AS1 in human cancers

EGFR-AS1 was recently shown to be upregulated in diverse types of cancers, including lung cancer (37–40), cervical cancer (41, 42), glioma (43, 44), bladder cancer (45, 46), kidney cancer (47, 48), head and neck cancer (49–51), gastric cancer (52), colorectal cancer (53), liver cancer (54), and uterine cancer (55) (**Figure 1B**). Numerous studies have indicated that high EGFR-AS1 expression is closely correlated with multiple clinical characteristics, including tumor size, clinical stage, vascular invasion, portal vein thrombosis, cumulative recurrence (CR), lymph node metastasis, overall survival (OS), disease-free survival (DFS), and recurrence-free survival (RFS) (**Table 1**). More importantly, EGFR-AS1 participates in cancer progression *via* regulation of biological processes, such as cell proliferation, migration, invasion, and even drug response (**Table 2**). In this review, we mainly illustrated the understanding of EGFR-AS1 in terms of its expression, correlation with clinicopathological characteristics, biological roles, and relevant mechanisms.

**Table 1:** EGFR-AS1 expression and clinical characteristics in cancers.

Disease type	Expression	Clinical characteristics	Ref.
lung cancer	overexpression	CR, OS, tumor size, and clinical stage	(37–40)
cervical cancer	overexpression	/	(41, 42)
glioma	overexpression	/	(43, 44)
bladder cancer	overexpression	OS, unfavorable tumor size, stages, grades, and lymph node metastasis	(45, 46)
renal cancer	overexpression	OS, RFS, DFS, tumor size, Fuhrman grade, TNM stage, erlotinib resistance, and distant metastasis	(47, 48)
head and neck cancer	overexpression	/	(49–51)
gastric cancer	overexpression	tumor size	(52)
colorectal cancer	overexpression	OS, tumor grade, tumor status, lymph node metastasis, and vascular invasion	(53)
liver cancer	overexpression	portal vein thrombosis, and lymph metastasis	(54)
uterine cancer	overexpression	/	(55)

**Table 2:** Functions and mechanisms of EGFR-AS1 in cancers.

Disease type	Role	Cell lines	Functions	Related mechanisms	Ref.
lung cancer	tumor promoter	A549, NCI-H460, NCI-H1299, NCI-H358, HCC827, NCH-H23, and NCI-H1650	cell proliferation, chemotherapy resistance, invasion, and stemness	miR-524-5p, DRAM1, miR-223, IGF1R, AKT, HIF2A, FOXP3, and Notch1	(34–37)
cervical cancer	tumor promoter	SiHa, CaSki, ME-180, and C4-1	cell proliferation, migration, and invasion	H3K27ac, miR-2355-5p, ACTN4, and WNT pathway	(38, 39)
glioma	tumor promoter	U87, U251, MG, and T98 G	cell migration, invasion, apoptosis, and drug resistance	miR-133b, and RACK1	(40, 41)
bladder cancer	tumor promoter	HT-1197, 5637, and T24	cell proliferation, and invasion	miR-381, and ROCK2	(42, 43)
renal cancer	tumor promoter	786O, OSRC-2, RCC4, ACHN, A498, and KETR-3	cell proliferation, drug sensitivity, and invasion	HuR, and EGFR	(44, 45)
head and neck cancer	tumor promoter	KYSE-30, EC109, NCC-HN19, NCC-HN64, NCC-HN1, and NCC-HN43	cell invasion, and migration	miR-145, and ROCK1	(46–48)
gastric cancer	tumor promoter	SGC7901, BGC823, MGC803, and MKN-28	cell proliferation	EGFR, and PI3K/AKT pathway	(49)
colorectal cancer	tumor promoter	/	/	miR-133b, EGFR, and STAT3	(50)
liver cancer	tumor promoter	SMMC-7721, LM-9, Huh-7, and HepG2	cell invasion, and proliferation	EGFR	(51)
uterine cancer	tumor promoter	SK-LMS-1, and SK-UT-1	T-cell infiltration, and immune escape	EGFR, MYC, and PD-L1	(52)

## Lung cancer

Several studies reported that EGFR-AS1 was overexpressed in lung tissues and cell lines (A549, NCI-H460, NCI-H1299, NCI-H358, HCC827, NCH-H23, and NCI-H1650). High expressed EGFR-AS1 was strongly associated with poor CR and OS, large tumor size, and advanced clinical stage. In addition, EGFR-AS1 was functionally proven to enhance the proliferation, chemotherapy resistance, invasion, and stemness abilities of lung cancer cells and the tumor growth of mouse xenografts (37–40).

## Cervical cancer

EGFR-AS1 expression was increased in cervical cancer tissues and different cell lines. Previous research indicated that EGFR-AS1 contributes to the progression of cervical cancer by promoting the proliferation, and invasion of SiHa and CaSki cells (41, 42).

## Glioma

In glioma, EGFR-AS1 expression was elevated in tissues and U87, U251, MG, and T98 G cells. EGFR-AS1 exerts its oncogenic roles through the promotion of cell migration, invasion, apoptosis, and drug resistance in U87 and U251 cells and through the promotion of xenograft growth (43, 44).

## Bladder cancer

High EGFR-AS1 levels were observed in bladder cancer HT-1197, 5637, and T24 cells as well as tissues. Increased EGFR-AS1 expression indicated a poor patient prognosis and unfavorable features in terms of tumor size, stages, grades, and lymph node metastasis. Functionally, EGFR-AS1 facilitates cell proliferation and invasion and tumor growth and metastasis, thus promoting bladder cancer progression (45, 46).

## Renal cancer

EGFR-AS1 overexpression was also discovered in renal cancer tissues and 786O, OSRC-2, RCC4, ACHN, A498, and KETR-3 cells. A high level of EGFR-AS1 was related to unfavorable features in terms of tumor size, Fuhrman grade, TNM stage, erlotinib resistance, distant metastasis, OS, RFS, and DFS. Additionally, EGFR-AS1 stimulated the proliferation, erlotinib resistance, and invasion of 786-O and A498 cells and lung metastasis of renal cancer cells in mice (47, 48).

## Head and neck cancer

Multiple studies have revealed the upregulation of EGFR-AS1 in the tumor tissues and cells of head and neck cancer, including oral squamous cell carcinoma and esophageal squamous cell carcinoma (ESCC) (49–51). EGFR-AS1 was also demonstrated to promote the invasion and migration of esophageal squamous cell carcinoma cells (KYSE-30 and EC109) and thus achieved its pro-oncogenic effect (49).

## Other cancers

In addition, EGFR-AS1 expression was upregulated in gastric cancer tissues and SGC7901, BGC823, MGC803, and MKN-28 cells. High EGFR-AS1 expression was remarkably associated with larger tumor size and promoted MGC803 and SGC-7901 cell proliferation as well as mouse tumor growth (52). In colorectal cancer, increased EGFR-AS1 levels reflect adverse patient features in terms of tumor grade, tumor status, lymph node metastasis, vascular invasion, and outcomes (53). EGFR-AS1 overexpression was also discovered in liver cancer tissues and a various of HCC cell lines and was closely correlated with lymph metastasis (54). *In vivo* and *in vitro* experiments validated that EGFR-AS1 promotes the invasion and proliferation of Huh-7 liver cells, resulting in the development of liver cancer (54). Additionally, EGFR-AS1 was upregulated in uterine cancer SK-LMS-1 and SK-UT-1 cells as well as tissues. EGFR-AS1 exhibited an oncogenic effect in uterine cancer by suppressing T-cell infiltration and motivating immune escape and tumor growth (55).

## The mechanism of EGFR-AS1 in human cancers

Multiple mechanistic studies have reported that lncRNAs function as tumor promoters through the regulation of a series of biological processes of cancers, such as cell proliferation, apoptosis, invasion and migration (22, 24, 28, 59, 60). In this section, we summarize the major biological mechanisms of EGFR-AS1 during cancer progression.

**Figure 2:**
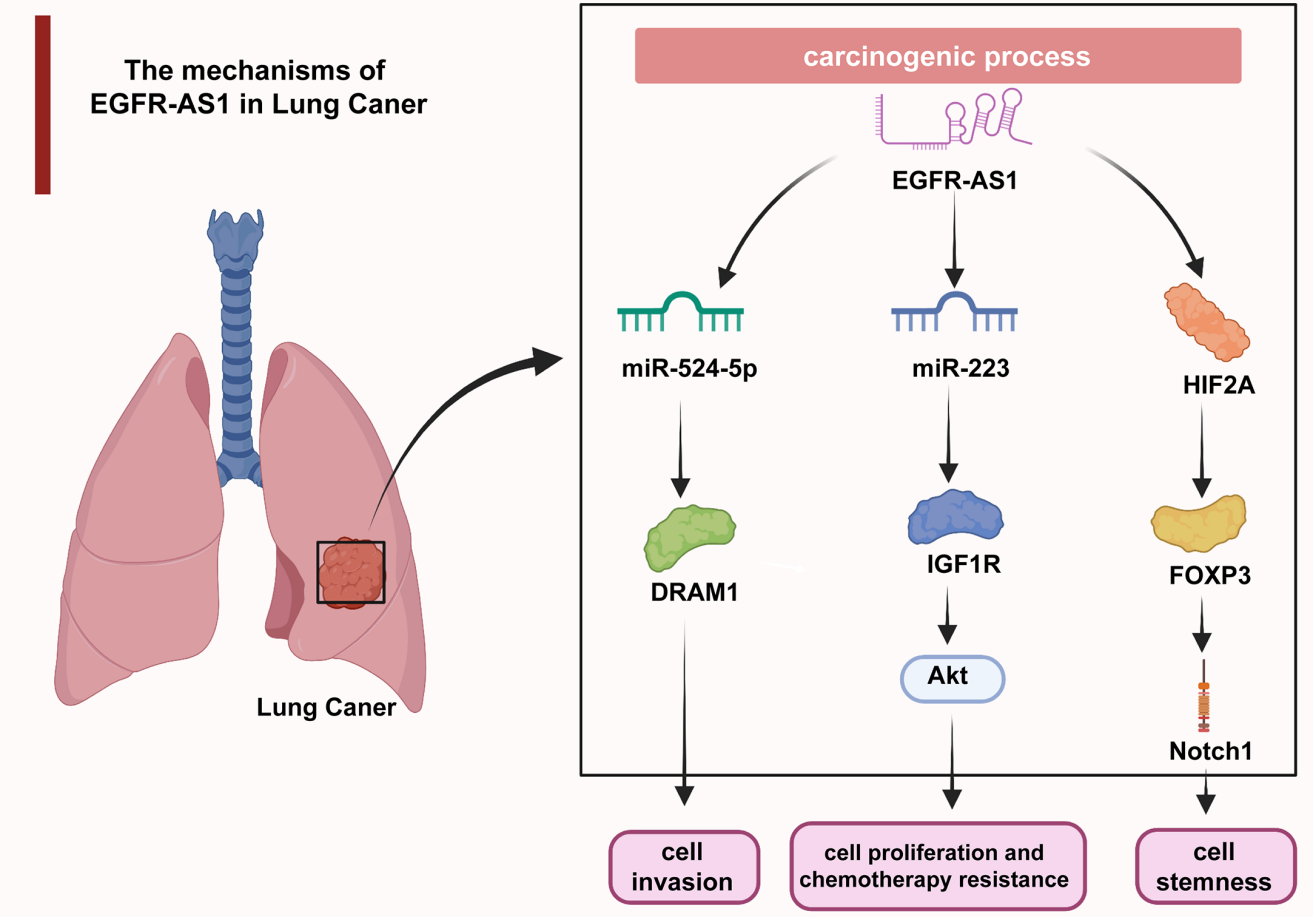
In lung cancer, EGFR-AS1 enhanced cell proliferation, chemotherapy resistance, invasion, and stemness *via* the miR-524-5p/DRAM1 axis, miR-223/IGF1R/AKT signaling pathway, and HIF2A/FOXP3/Notch1 axis.

Uncontrolled cell proliferation causes malignant transformation and ultimately tumorigenesis, which has long been a hotspot in cancer research (61–65). Additionally, aberrantly activated cell migration and invasion are responsible for tumor expansion into the adjacent tissues, which accounts for more than 90% of cancer-related deaths (66–69). The understanding of the molecular mechanisms underlying dysregulated cellular processes may shed light on the improvement of cancer management (70–74). As revealed by multiple studies, EGFR-AS1 participates in the mediation of diverse cellular activities through interactions with its target molecules. In lung cancer, EGFR-AS1 inhibited miR-524-5p and rescued DRAM1 expression and therefore promoted the invasion of HCC827 and NCI-H1650 cells (37). EGFR-AS1 was also indicated to enhance the proliferation and chemotherapy resistance of NCI-H1299 and NCI-H358 cells *via* the miR-223/IGF1R/AKT signaling pathway (39). In addition, previous research reported that nicotine-derived nitrosamine ketone (NNK) downregulated EGFR-AS1 levels and then elevated HIF2A and FOXP3 expression and promoted Notch1-mediated enhancement of cancer cell stemness (40) (**Figure 2**). In cervical cancer, H3K27 acetylation-activated EGFR-AS1 interacts with miR-2355-5p and activates the ACTN4-mediated WNT pathway to promote the proliferation, migration, and invasion of SiHa and CaSki cells (42). It was also shown in glioma U87 and U251 cells that EGFR-AS1 promotes cell migration and invasion by sponging miR-133b to facilitate RACK1 expression (43). In bladder cancer, EGFR-AS1 accelerates cell proliferation and invasion in the T24 and 5637 cell lines by increasing the expression of EGFR (45). EGFR-AS1 also sponges microRNA-381 to elevate ROCK2 levels, which promotes bladder cancer HT-1197 cell invasion and migration (46). Moreover, EGFR-AS1 enhances the cell proliferation and invasion abilities of renal cancer 786-O and A498 cells through the enhancement of HuR-mediated EGFR expression (48). In esophageal squamous cell carcinoma, EGFR-AS1 combines with miR-145 and increases ROCK1 expression to increase the rates of KYSE-30 and EC109 cell invasion and migration (49). EGFR-AS1 was also proven to stabilize EGFR expression *via* the EGFR-dependent PI3K/AKT pathway and to facilitate the proliferation of MGC803 and SGC-7901 gastric cancer cells (52). In colorectal cancer, EGFR-AS1 exerts a pro-oncogenic effect through the miRNA-133b/EGFR/STAT3 axis (53). EGFR-AS1 also decreases EGFR levels to enhance the invasion and proliferation abilities of Huh-7 liver cancer cells (54). Moreover, EGFR-AS1 combines with EGFR to stimulate MYC and subsequent PD-1 expression, achieving uterine cancer cell proliferation (55).

## Clinical application of EGFR-AS1 in human cancers

**Figure 3:**
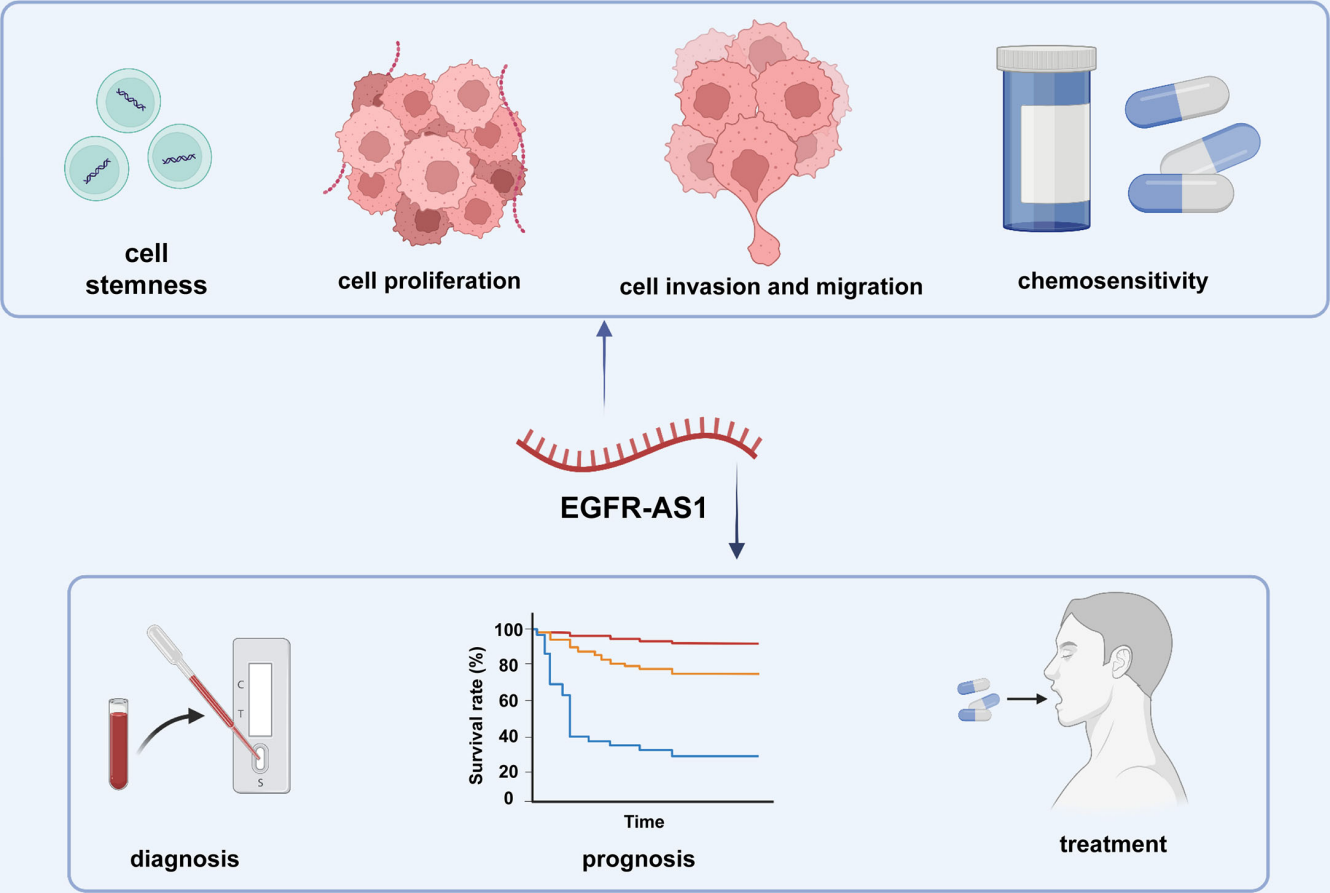
The clinical applications of EGFR-AS1.

Mounting evidence indicates that lncRNAs play pivotal roles in the different stages of cancer progression and possess considerable potential for clinical diagnosis, prognosis evaluation, and even treatment (23, 75–77) (**Figure 3**). Concerning its broad participation in the regulation of diverse cellular processes, EGFR-AS1 cloud be a promising novel biomarker for disease diagnosis, treatment and prognosis prediction. For example, given the overexpression of EGFR-AS1 in lung cancer patient plasma, plasma EGFR-AS1 was regarded as a noninvasive marker for cancer diagnosis as well as an independent prognostic predictor for the CR and OS of lung cancer patients (39). Xu et al. further indicated that EGFR-AS1 expression has an inverse relationship with the response to cisplatin and gemcitabine (39). EGFR-AS1 was also experimentally validated by Nath A et al. as a predictive marker of lung cancer patient response to anti-EGFR drugs such as erlotinib (38). Similarly, high EGFR-AS1 levels could differentiate bladder cancer tissue from adjacent normal tissues with a high AUC value of 0.845. Kaplan–Meier analysis also suggested that increasing EGFR-AS1 expression in bladder cancer was markedly associated with poor outcomes in terms of factors such as DFS and OS (45). As an independent prognostic factor for renal cancer patients, EGFR-AS1 was demonstrated to regulate sensitivity to the EGFR inhibitor erlotinib/Tarceva (ERLO) (47, 48). Survival curves for esophageal squamous cell carcinoma showed that the 5-year survival rate of patients in the EGFR-AS1 overexpression group was distinctly decreased (49). In oral squamous cell carcinoma, EGFR-AS1 knockdown was proven to reverse patient resistance to tyrosine kinase inhibitors (50). Similar results regarding the potential of EGFR-AS1 for disease diagnosis and prognosis were found in colorectal and liver cancer (53, 54).

## Conclusions

EGFR-AS1 was found to be overexpressed in diverse cancers, including lung cancer, cervical cancer, glioma, bladder cancer, kidney cancer, head and neck cancer, gastric cancer, colorectal cancer, liver cancer, and uterine cancer. Upregulation of EGFR-AS1 was reported to be closely correlated with unfavorable clinicopathological characteristics such as tumor stage and distant metastasis as well as a poor prognosis in patients with multiple cancers. *In vitro* and *in vivo* research elucidated the molecular mechanisms of EGFR-AS1 in cancer-related biological processes, including cell proliferation, invasion, and migration, which mainly involve interaction with its target molecules. Regarding the exploration of its clinical value, EGFR-AS1 was validated to function as a sensitive indicator of cancer diagnosis, prognosis, and treatment response in several cancer types. Further studies clarifying the in-depth mechanisms of EGFR-AS1 in cancer progression and validating the efficacy and safety of EGFR-AS1 application in cancer management are warranted.

## Author contributions

LL and KS designed and guided the study. DZ and XO wrote and edited the manuscript. YZ and XY helped with reference collection. All authors contributed to the article and approved the submitted version.
